# Safety and efficacy of the addition of simvastatin to cetuximab in previously treated *KRAS* mutant metastatic colorectal cancer patients

**DOI:** 10.1007/s10637-015-0285-8

**Published:** 2015-09-19

**Authors:** J. M. Baas, L. L. Krens, A. J. ten Tije, F. Erdkamp, T. van Wezel, H. Morreau, H. Gelderblom, H. J. Guchelaar

**Affiliations:** Department of Clinical Oncology, Leiden University Medical Center, Albinusdreef 2, P.O. Box 9600, 2300 RC Leiden, The Netherlands; Department of Clinical Pharmacy and Toxicology, Leiden University Medical Center, Albinusdreef 2, 2300 RC Leiden, The Netherlands; Department of Clinical Oncology, Amphia Hospital, Langendijk 75, 4819 EV Breda, The Netherlands; Department of Clinical Oncology, Orbis Medical Center, Dr. H. van der Hoffplein 1, 6162 BG Sittard–Geleen, The Netherlands; Department of Pathology, Leiden University Medical Center, Albinusdreef 2, 2300 RC Leiden, The Netherlands

**Keywords:** *KRAS*, Colorectal cancer, Cetuximab, Statin

## Abstract

*Introduction* Cetuximab is registered for use in colorectal cancer (CRC) patients with *RAS* wild-type tumours only. Simvastatin blocks the mevalonate pathway and thereby interferes with the post-translational modification (prenylation) of KRAS. We hypothesize that the activitated KRAS pathway in *KRAS* mutant tumors can be inhibited by simvastatin rendering these tumors sensitive to the EGFR inhibitor cetuximab. *Methods* A Simon two-stage, single-arm, phase II study was performed to test the efficacy and safety of the addition of simvastatin to cetuximab in patients with a *KRAS* mutation in their CRC tumour who were previously treated with fluoropyrimidine, oxaliplatin and irinotecan based regimens. The primary endpoint was to test the percentage of patients alive and free from progression 12.5 weeks after the first administration of cetuximab. Our hypothesis was that at least 40 % was free from progression, comparable to, though slightly lower than in *KRAS* wild-type patients. *Results* Four of 18 included patients (22.2 %) were free from progression at the primary endpoint time. The time to progression in these 4 patients ranged from 20.3 to 47 weeks. *Conclusion* Based on the current study we conclude that the theoretical concept of KRAS modulation with simvastatin was not applicable in the clinic, as we were not able to restore sensitivity to cetuximab in CRC patients harbouring a somatic *KRAS* mutation.

## Introduction

Each year over 940.000 patients are diagnosed with colorectal cancer (CRC) world-wide and over 500.000 people die of this disease [1]. In patients with advanced or metastatic colorectal treatment with monoclonal antibodies directed against the epidermal growth factor receptor (EGFR), cetuximab and panitumumab are proven to be active after failing fluoropyrimidine, oxaliplatin and irinotecan based regimens, though only in patients with tumours without a mutation in the *KRAS* [2, 3] or more recently *RAS* gene [4]. This led to the question whether increased activation of KRAS signaling by *KRAS* mutations can be modulated, thereby making *KRAS* mutated tumours sensitive to EGFR inhibitor therapy. One possible target for modulation is the mevalonate pathway, as we have previously discussed [5].

The mevalonate pathway is a metabolic cascade with various end-products including cholesterol. Other end-products are farnesyl and geranylgeranyl moieties (C15 and C17), both essential for posttranslational prenylation of the RAS protein and its association with the cytoplasmic membrane, and thereby activation of the RAS protein. By using HMG-CoA reductase inhibitors not only the synthesis of cholesterol is inhibited, but also the formation of C15 and C17, thereby inhibiting posttranslational modification of RAS [5, 6]. By blocking the mevalonate pathway in CRC patients with *KRAS* mutated tumours, the activated KRAS pathway might be inhibited. This would theoretically lead to increased sensitivity to cetuximab, potentially comparable to tumours with wild-type *KRAS*.

This single-arm, phase II study was designed to test the safety and efficacy of the addition of simvastatin to cetuximab in patients with a *KRAS* mutation in their tumour who were previously treated with fluoropyrimidine, oxaliplatin and irinotecan based regimens.

## Methods

### Patients

Eligible patients had advanced or metastatic colorectal cancer with a mutation in codon 12, 13 or 61 of the *KRAS* gene (either on tissue of the primary tumour or of a metastasis), after failing fluoropyrimidine, oxaliplatin and irinotecan based regimens, or after failure of oxaliplatin based therapy in patients who cannot be treated with irinotecan.

Other eligibility criteria included age 18 years or older, written informed consent, World Health Organisation (WHO) performance score of 0 to 2 and progression of disease in the past 3 months prior to inclusion. Exclusion criteria included symptomatic brain metastases, previous treatment with EGFR inhibitors and history of toxicity during statin.

The study protocol was approved by the Ethics Committees of all participating hospitals.

### Study design

This phase II, single-arm, multi-center study was performed using a Simon two-stage design [7]. In the first stage, 15 patients were included, followed by an interim analysis. Results of this analysis would determine whether the combination of simvastatin and cetuximab may have clinical benefit in this group of CRC patients, thus justifying the second stage and including up to 41 patients.

### Treatment schedule

Cetuximab was first administered at least one week after start of simvastatin therapy. The initial cetuximab dose was 400 mg/m^2^ with subsequent weekly infusions of 250 mg/m^2^. Pretreatment with an antihistamine and a corticosteroid was mandatory before the first infusion of cetuximab and recommended for all subsequent infusions.

Simvastatin 80 mg orally once daily was started at start of study participation. This dose was chosen taken into consideration the need for continuous administration of the statin during the entire study, inhibitory effect on the mevalonate pathway and tolerability. Statins in cancer therapy have been studied in clinical trials in solid [8–18] and haematologic [19–21] malignancies, both as monotherapy as well as additional to chemotherapy. Statin doses from 20 mg/day up to 35 mg/kg/day were used, with only continuous use of statins when dosed at a maximum of 80 mg/day. Since the aim of this study is to modulate KRAS during the entire treatment with cetuximab and therefore a continuous exposure to simvastatin is needed, a dose of 80 mg/day was selected in order to obtain maximum effect while minimizing the risk of toxicity.

Treatment was continued until progression of disease, clinical signs of progression, unacceptable toxicity or cetuximab toxicity requiring withholding of more than 2 subsequent infusions.

Tumour response was every 6 weeks using CT-scans and according to Response Evaluation Criteria In Solid Tumors (RECIST) version 1.1. Scans of patients free from progression at time of primary endpoint were centrally reviewed.

### Endpoints

Primary objective was to test the percentage of patients alive and free from progression and alive at 12.5 weeks after the first administration of cetuximab. Our hypothesis was that at least 40 % of patients was free from progression, comparable to though slightly lower than in *KRAS* wild-type patients [2].

Secondary objectives were to investigate overall survival (OS), objective response rate (ORR), progression free survival (PFS), and safety of simvastatin combined with cetuximab in this population and to evaluate the correlation between skin toxicity and response to treatment. Exploratory endpoints were to investigate the role of cholesterol as a biomarker during this treatment and whether *PIK3CA* status correlates with response to cetuximab in this population.

### Mutational analysis

*KRAS* mutational status was reconfirmed centrally, testing for the 7 most frequent mutations in codon 12 and 13 as described in detail elsewhere [22]. In addition, we tested for the 3 most common mutation in the *PIK3CA* gene; in exon 9 (c.1624G>A (p.E542K) and c.1633G>A (pE545K)) and exon 20 (c.3140A>G (p.H1047R)). Though *KRAS* and *BRAF* mutations are known to be mutually exclusive [23], we did test for the activating hotspot mutation p.V600E.

### Statistics

Sample size was chosen based on previous published data of CRC patients with *KRAS* wild-type tumours treated with cetuximab [2], aiming for a at least 40 % of patients free from progression at 12.5 weeks after start of cetuximab treatment in patients with *KRAS* mutant type tumours (i.e., slightly lower than the effect in *KRAS* wild-type patients). Combined with an alpha of 0.05 and a power of 0.80, an interim size of 15 and a total sample size of 46 patients were required. An interim analysis was to be performed after the inclusion of 15 evaluable patients. Only when at least 40 % (i.e., 6 patients) were free from progression at the 12.5 weeks, another 31 patients would be enrolled during the second stage of the study.

## Results

### Patients

During the first stage of the study 18 instead of 15 patients were enrolled to account for patients that were thought to unevaluable for the primary endpoint. Baseline characteristics are listed in Table 1. None of the patients were using statins prior to inclusion.

**Table 1:** Baseline characteristics

Age – years	
Mean	62
Range	52–75
Gender – *n* (%)	
Male	13 (72)
Female	5 (18)
WHO performance score – *n* (%)	
0	13 (72)
I	5 (18)
Site of primary tumour – *n* (%)	
Colon	12 (67)
Rectum	6 (33)
Prior lines of chemotherapy – *n* (%)	
1	2
2	15
3	1
Prior surgery – *n* (%)	13 (72)
Prior radiotherapy – *n* (%)	4 (22)

### Efficacy

Four of 18 patients were free from progression at the primary endpoint time, therefore the percentage of patients alive and free from progression 12.5 weeks after the first administration of cetuximab was 22 %. The time to progression in these 4 patients ranged from 20.3 to 47 weeks. Drug exposure to simvastatin and cetuximab was equal for all patients.

**Fig. 1:**
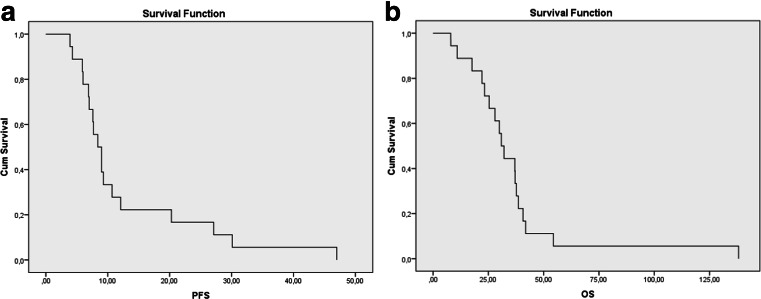
**a** Progression free survival in weeks for the addition of simvastatin to cetuximab in CRC patients failing standard therapy. **b** Overall survival in weeks for the addition of simvastatin to cetuximab in CRC patients failing standard therapy

Figure 1 shows progression free (panel A) and overall survival (panel B). Median PFS was 9 weeks (mean 12.9 weeks, range 3.9–47 weeks). Median OS was 31.5 weeks (mean 36.3, range 8–138.1). The ORR was 6 % (partial remission in 1 patient). A true relation between skin toxicity and efficacy of treatment was not observed in this study though this may (partly) be due to the low number of patients and due to the improved knowledge of the efficacy of pre-emptive skin toxicity management.

### Safety

Main symptoms and adverse events reported on study reported were fatigue (*n* = 11), acne (*n* = 10) and rash (*n* = 6). Myopathy was not reported. Three patients had elevation of creatine kinase (CK) levels on study (grade 4 in one patient). Skin toxicity occurred in 10 patients; the worst grade of acneiform rash was grade 3 in one patient, grade 2 in 4 patients and grade 1 in the remaining 5 patients. One patient experienced a severe (i.e., grade 3) allergic reaction during the first infusion of cetuximab. One of the serious adverse events did precede the death of a participant. Upon the scheduled laboratory examination severe elevation of liver enzymes were observed. Rhabdomyolysis was considered, (though on study CK levels were below 3.000 U/l) and so was progression of liver metastases. Study medication was interrupted immediately, however the patient’s situation did not improve and it was decided to terminate study participation permanently. Specific SNPs associated with increased risk of statin-induced myopathy (i.e., *SLCO1B1* variants [24]) were considered though none were identified in this patient. The patient deceased few weeks later. Post-mortem examination did not occur.

### Exploratory endpoints

All patients showed cholesterol reduction, ranging from a maximum reduction of 0.8 to 64.4 %. The percentage of cholesterol reduction did not correlate with progression free survival.

Tumour tissue of 15 patients was available for central review. Table 2 shows mutational status of *KRAS* and *PIK3CA* per patient. Of the 4 patients responding to treatment, 3 had a *KRAS* mutation in codon 12 and 1 had a *PIK3CA* mutation. As expected all patients were *BRAF* wild-type.

**Table 2:** *KRAS* and *PIK3CA* mutational status per patient

Study number	*KRAS* mutation	*PIK3CA* mutational status
1	G12D	Wild-type
2	G12V	Wilde-type
3	G12V	Wild-type
4	G12C	Wild-type
5	G12V	Wild-type
6	G12S	Wild-type
7	*missing*	*Missing*
8	G12V	Wild-type
9	G13D	Wild-type
10	G13D	Wild-type
11	G12D	Wild-type
12	G12D	Wild-type
13	*missing*	*Missing*
14	G12V	Wild-type
15	G12A	Wild-type
16	G12A	Wild-type
17	G13D	Mutation in exon 9
18	G12D	Mutation in exon 9

## Discussion

To our knowledge, this is the first clinical trial testing the addition of simvastatin to cetuximab monotherapy in CRC patients harbouring a *KRAS* mutation in tumour tissue as an attempt to restore cetuximab sensitivity. While it was remarkable to notice a durable progression free survival in 4 patients, the interim analysis showed that the predefined criteria to proceed to the second stage of this study were not reached. Therefore, the current study suggests that high dose simvastatin does not render cetuximab sensitivity in *KRAS* mutant CRC.

Statines are one of several potential agents to modulate KRAS signaling, as we have previously reviewed [5]. The current study is not the first to hypothesize on statins and their inhibitory effect on the activity of RAS and its downstream pathway. However, all but one previous reports include only preclinical data. For example, lovastatin and simvastatin inhibit downstream activity in breast cells with mutated *HRAS*, possibly by inhibiting membrane localization of HRAS. The effect was reversed when adding farnesyl pyrophosphate, indicating the effect was related to prenylation of RAS [25]. More recently, simvastatin was shown to restore cetuximab resistance in vitro and in vivo [26]. Based on these results, one might wonder whether the negative outcome of the current study would have been different if using higher doses of simvastatin. However, preclinical data showed a significant reduction in cell growth of *KRAS* mutant CRC cell lines using 0.2 μM simvastatin, the equivalent of 2 mg/kg/day in humans [26]. Moreover, in cardiovascular disease the registered dose of 80 mg of simvastatin is significantly lowers cholesterol serum levels. It is reasonable that this dose will also affect the formation of the C15 and C17 groups and subsequently the prenylation of the KRAS protein. Furthermore, we question whether higher doses will be feasible in terms of safety.

A recent study of Lee et al. tested the efficacy of the addition of the same dose of simvastatin (i.e., 80 mg once daily) to cetuximab and irinotecan in *KRAS* mutant CRC patients failing prior oxaliplatin, fluoropyrimidine and irinotecan based therapy [27]. The initially reported PFS and OS (median 7.6 months and 12.8 months respectively) were considerably higher than historical results in chemotherapy refractory CRC patients with *KRAS* mutated tumours [28] and chemotherapy refractory CRC patients in general [29–31]. Moreover, these results were in contrast with our findings. However, a recent erratum published by this group showed that initial survival data were incorrect [32]. The corrected PFS and OS are in line with our results, providing no evidence for a modulating effect of simvastatin on the *KRAS* mutant phenotype.

The majority of patients had a *KRAS* mutation in codon 12 and only 3 in codon 13. It has been reported that tumours harbouring a G13D mutation in the *KRAS* gene might be sensitive to EGFR-inhibitors [33]. Moreover, none of our patients had a *PIK3CA* mutation in exon 20, while these might also be more likely to be sensitive to EGFR-inhibitors, contrary to mutations in exon 9 [34]. However, of the 4 patients who were free from progression at time of the primary endpoint only one patients had a G13D mutation in the *KRAS* gene and none had a *PIK3CA* mutation in exon 20.

## Conclusion

Based on the current study we conclude that the concept of *KRAS* modulation with simvastatin was not applicable in the clinic. Similar results were recently demonstrated in this population treated with panitumumab and simvastatin [35]. Better treatment strategies are needed for this patient population.
